# The Role of the Family and Community Nurse in Improving Quality of Life and Optimizing Home Care Post-COVID: A Systematic Review with Meta-Analysis

**DOI:** 10.3390/nursrep15120415

**Published:** 2025-11-26

**Authors:** Angelo Cianciulli, Emanuela Santoro, Nicole Bruno, Savino Quagliarella, Salvatore Esposito, Roberta Manente, Biagio Santella, Rosaria Flora Ferrara, Antonietta Pacifico, Gianluigi Franci, Giovanni Boccia

**Affiliations:** 1 Department of Medicine, Surgery and Dentistry “Scuola Medica Salernitana”, University of Salerno, 84081 Salerno, Italy; ancianciulli@unisa.it (A.C.); n.bruno5@studenti.unisa.it (N.B.); s.quagliarella@studenti.unisa.it (S.Q.); s.esposito146@studenti.unisa.it (S.E.); bsantella@unisa.it (B.S.); rosferrara@unisa.it (R.F.F.); apacifico@unisa.it (A.P.); gfranci@unisa.it (G.F.); gboccia@unisa.it (G.B.); 2 San Giovanni di Dio e Ruggi d’Aragona University Hospital, 84081 Salerno, Italy; manente392@gmail.com; 3 Microbiology and Virology Unit, San Giovanni di Dio e Ruggi d’Aragona University Hospital, 84131 Salerno, Italy; 4 Integrated Care Department of Health Hygiene and Evaluative Medicine, San Giovanni di Dio e Ruggi d’Aragona University Hospital, 84131 Salerno, Italy; 5 Hospital and Epidemiological Hygiene Unit, San Giovanni di Dio e Ruggi d’Aragona University Hospital, 84131 Salerno, Italy

**Keywords:** family nurse, community nursing, post-COVID care, quality of life, home care, systematic review, meta-analysis

## Abstract

**Background/Objectives**: The COVID-19 pandemic accelerated the shift toward community- and home-based care models. Within this transformation, Family and Community Nurses (FCNs) have become key in bridging hospital and primary care, supporting continuity, self-care, and quality of life (QoL). Despite increasing recognition, evidence on FCN-led interventions remains fragmented. This systematic review and meta-analysis aimed to synthesize evidence on the impact of FCN interventions on QoL and clinical outcomes in post-COVID and people living with chronic conditions managed in community and home settings. **Methods**: Following PRISMA 2020 guidelines, we searched PubMed, Scopus, CINAHL, PsycINFO, Embase, and Cochrane Library (January 2020–November 2024). Eligible studies were randomized controlled trials evaluating FCN-led interventions. Primary outcomes were QoL (measured with validated tools) and glycemic control (HbA1c). Secondary outcomes included hospital readmissions, anxiety, depression, and self-care abilities. Risk of bias was assessed using the Cochrane RoB2 tool for randomized controlled trials. Random-effects meta-analyses were performed, with heterogeneity evaluated by I^2^. The protocol was prospectively registered in PROSPERO (CRD42024567890) before data extraction. **Results**: Seventy-one studies (*n* = 19,390) were included. Interventions comprised home visits, telehealth, patient education, and case management. Pooled analyses demonstrated significant improvement in QoL (SMD 0.34, 95% CI 0.18–0.50) and reduction in HbA1c (−0.47%, 95% CI −0.69 to −0.25). FCN interventions also reduced hospital readmissions (RR 0.74, 95% CI 0.62–0.89) and improved mental health outcomes. Most studies were judged at low to moderate risk of bias. **Conclusions**: FCN-led interventions significantly enhance QoL, mental health, and clinical outcomes while reducing hospital readmissions. These findings highlight the strategic importance of integrating FCNs into community-based healthcare models.

## 1. Introduction

The COVID-19 pandemic has underscored the vulnerability of healthcare systems worldwide, highlighting the need for resilient community- and home-based care models [1,2]. In this scenario, Family and Community Nurses (FCNs) have become pivotal in bridging gaps between hospital and primary care, addressing both acute and long-term consequences of COVID-19, as well as pre-existing chronic conditions [3,4]. FCNs play a central role in health promotion, disease prevention, continuity of care, and the empowerment of patients and caregivers, particularly in contexts where health services are fragmented [5,6,7].

Although their contribution has been acknowledged in policy frameworks and health reforms (e.g., the Italian Ministerial Decree 77/2022, which defines territorial standards for community care) [8], the empirical evidence on the actual effectiveness of FCN-led interventions remains heterogeneous. Studies vary in terms of populations, outcomes assessed, and methodological rigor, creating uncertainty for policymakers and practitioners.

For the purposes of this review, an “FCN-led intervention” was operationally defined as any programme in which the primary provider was a Family or Community Nurse (or an equivalent role depending on jurisdiction), including nurses trained in community health, public health nursing, or case management. Multidisciplinary or mixed-provider interventions were included only when the FCN had a central, clearly identifiable coordinating role. Studies in which the principal interventionist was another professional (e.g., therapists, social workers, physicians) without FCN leadership were excluded.

The recognition of FCNs as cornerstones of territorial health systems calls for a robust evidence synthesis that goes beyond anecdotal reports or narrative reviews. Previous overviews have suggested potential benefits of eHealth- and nurse-led interventions in chronic disease management [9,10], but no comprehensive systematic review has specifically addressed the impact of FCNs on post-COVID care and home-based health outcomes. Considering the increasing demand for integrated care and the persistent burden of long COVID symptoms, synthesizing available evidence is crucial to inform service design, resource allocation, and professional training.

The primary objective of this study was to evaluate the effectiveness of FCN-led interventions on patients’ QoL and clinical outcomes in post-COVID and chronic care contexts. The secondary objective was to examine their impact on hospital readmissions, mental health outcomes, and self-care behaviors. We hypothesized that FCN-led interventions would be associated with measurable improvements in QoL and clinical parameters, alongside reductions in hospital utilization and psychological distress.

### Research Question

Based on the PEO/PICO framework, this review aimed to answer the following question: “Among community-dwelling individuals with chronic or post-COVID conditions, what are the effects of FCN-led interventions compared with usual care on clinical outcomes, patient-reported outcomes, and health service utilization?”.

## 2. Materials and Methods

### 2.1. Study Design and Setting

This systematic review and meta-analysis was conducted in accordance with the Preferred Reporting Items for Systematic Reviews and Meta-Analyses (PRISMA 2020) guidelines [11,12]. The protocol was prospectively registered in PROSPERO (ID: CRD42024567890), on 6 November 2024. In the final stage of the review, we introduced a minor deviation from the initial protocol: although observational studies were originally eligible, they were excluded from the quantitative synthesis due to substantial methodological heterogeneity and incomplete reporting, and only randomized controlled trials (RCTs) were meta-analysed. All deviations were documented to maintain full transparency The primary aim was to evaluate the effectiveness of Family and Community Nurse (FCN)-led interventions on quality of life (QoL) and clinical outcomes in patients receiving home- and community-based care, including those with post-COVID sequelae and chronic conditions.

### 2.2. Eligibility Criteria

#### 2.2.1. Studies Were Eligible if They Met the Following Criteria

- Population: adults (≥18 years) with post-COVID sequelae or chronic diseases (e.g., diabetes, COPD, heart failure).
- Studies were eligible if the intervention recipients were adults (≥18 years), including adult caregivers, even when the care recipient (e.g., pediatric patient) was under 18. This inclusion reflects the focus on FCN-led interventions delivered to adults in family and community contexts.
- Intervention/Exposure: structured FCN-led interventions such as home visits, telehealth, case management, patient education, or multidisciplinary coordination.
- Comparator: usual care or standard community/primary care without FCN involvement.
- Language: studies published in English, Italian, or Spanish.

A summary of eligibility criteria is presented in Table 1.

#### 2.2.2. Outcomes

- Primary outcomes: QoL measured with validated tools (e.g., EQ-5D, SF-36) and glycemic control (HbA1c).
- Secondary outcomes: hospital readmissions, psychological health (e.g., HADS, GAD-7), and self-care behaviors (e.g., Self-Care of Chronic Illness Inventory).

Study design: randomized controlled trials (RCTs) and observational cohort studies. Case reports, reviews, protocols, and qualitative studies were excluded.

Language and timeframe: English or Italian, published between January 2020 and November 2024.

### 2.3. Information Sources and Search Strategy

A comprehensive literature search was conducted in PubMed, Scopus, CINAHL, PsycINFO, Embase, and the Cochrane Library. The PubMed search strategy included combinations of controlled vocabulary (MeSH) and free-text terms such as “family nurse”, “community nursing”, “post-COVID”, “quality of life”, and “home care”. Boolean operators (AND/OR) and truncations were applied. Search strategies for all databases are reported in Supplementary S1. Reference lists of relevant reviews and included studies were manually screened to identify additional eligible publications. Language filters were applied from the outset in each database. Grey literature, conference abstracts, theses, and non-peer-reviewed materials were not included, as the review focused exclusively on peer-reviewed scientific publications.

**Figure 1:**
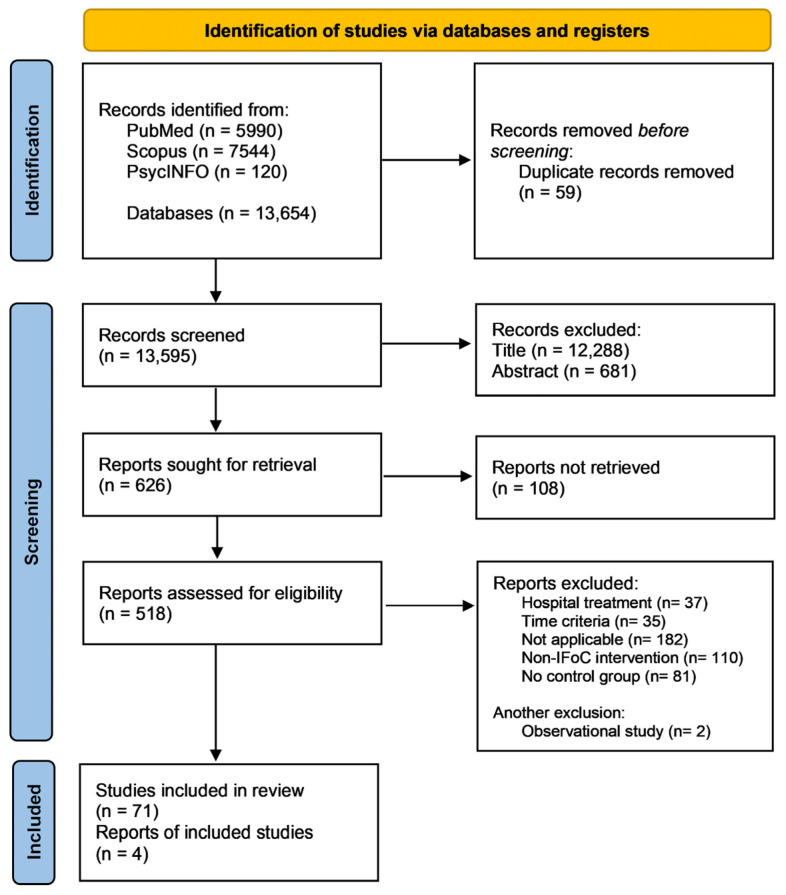
PRISMA flow diagram of the study identification, screening, eligibility assessment, and inclusion process.

All retrieved records were imported into Zotero software version. 7.0.26 [13]. Screening of titles and abstracts was independently performed by two reviewers (SQ, NB). Full texts of potentially relevant articles were retrieved and assessed for eligibility. Any disagreements were resolved through discussion, with a third reviewer (AC) serving as arbiter. The selection process was documented with a PRISMA flow diagram (Figure 1).

### 2.4. Data Extraction

Data from the included studies were extracted using a structured form that was developed and piloted by the review team to ensure consistency. Two reviewers (SQ, NB) independently performed data extraction, and a third reviewer (AC) verified all entries for accuracy and completeness.

For each study, we collected information on:

- Bibliographic details: author, year of publication, country, WHO region.
- Study design and setting: randomized controlled trial, cohort, or other eligible design; home-based, telehealth, or mixed interventions.
- Population characteristics: sample size, mean age, sex distribution, comorbidities.
- Intervention characteristics: content, intensity, frequency, and duration of the FCN-led intervention, including description of telemonitoring tools when applicable.
- Comparator: type and nature of usual care or control group.
- Outcomes and measures: primary and secondary outcomes assessed, instruments used (e.g., EQ-5D, SF-36, HbA1c, HADS, SC-CII), and timing of follow-up assessments.
- Results: effect estimates (means, proportions, relative risks, standard deviations, 95% confidence intervals), as well as narrative results when quantitative data were unavailable.

All extracted data were cross-checked to identify discrepancies. Differences were resolved by discussion, and when consensus could not be reached, arbitration was provided by a third reviewer (AC). To minimize transcription errors, double data entry was used for critical outcomes such as quality of life and HbA1c.

A summary template of the extracted fields is shown in Table 2.

A summary of the extracted domains is reported in Table 2, while the full extraction matrix for each included study is provided in Supplementary S2.

### 2.5. Risk of Bias Assessment

The methodological quality of randomized controlled trials (RCTs) was assessed using the revised Cochrane Risk of Bias 2.0 tool (RoB2) [14]. This instrument evaluates five domains of potential bias: (1) bias arising from the randomization process, (2) deviations from intended interventions, (3) missing outcome data, (4) measurement of outcomes, and (5) selection of the reported result. Each domain was judged as “low risk,” “some concerns,” or “high risk,” leading to an overall judgment for each trial. Two reviewers (SQ, NB) independently conducted the assessments, with discrepancies resolved by consensus and, if needed, by arbitration of a third reviewer (AC).

### 2.6. Data Synthesis and Statistical Analysis

Quantitative synthesis was undertaken when at least three studies reported the same outcome using comparable measures. For continuous outcomes such as quality of life (QoL), HbA1c, and anxiety/depression scores, pooled effect sizes were expressed as standardized mean differences (SMD) or mean differences (MD) with 95% confidence intervals (CIs). For dichotomous outcomes, including hospital readmissions, pooled relative risks (RR) with 95% CIs were calculated.

Meta-analyses were performed using a random-effects model (DerSimonian–Laird method) to account for potential between-study heterogeneity [15]. Statistical heterogeneity was assessed using the χ^2^ test and quantified with the I^2^ statistic, with thresholds of 25%, 50%, and 75% indicating low, moderate, and high heterogeneity, respectively. In addition, sensitivity analyses were conducted by excluding trials at high risk of bias and by performing subgroup analyses according to intervention modality (e.g., telehealth vs. home visits) and geographical region. Fixed-effects models were also computed as sensitivity checks to assess the robustness of pooled estimates. When essential data were missing, study authors were contacted by email to request additional information. If unavailable, missing standard deviations were imputed using established methods outlined in the Cochrane Handbook for Systematic Reviews of Interventions [15]. To evaluate the impact of imputed data, sensitivity analyses were performed comparing results with and without imputation. Publication bias was assessed by visual inspection of funnel plots for asymmetry and formally tested using Egger’s regression asymmetry test when ≥10 studies were available for a given outcome [16]. All statistical analyses were performed using R software version 4.3.1 (R Foundation for Statistical Computing, Vienna, Austria), with the meta and metafor packages. Quantitative pooling was performed only when ≥3 studies reported compatible effect measures (means, SDs, and sample sizes for continuous outcomes; or event counts for dichotomous outcomes). Publication bias (Egger’s test) was assessed only where ≥10 studies were available; otherwise, visual inspection of funnel plots was reported.

## 3. Results

### 3.1. Study Selection

The initial database search identified 13,654 records. After removing 59 duplicates, 13,595 titles and abstracts were examined. Of these, 12,288 were excluded at the title level and 681 at the abstract level, leaving 626 reports sought for retrieval. A total of 108 full-texts could not be retrieved, resulting in 518 studies assessed for eligibility. After full-text assessment, 445 studies were excluded for not meeting the inclusion criteria. Ultimately, 73 studies met the eligibility criteria; however, to ensure methodological homogeneity, two observational studies were excluded, and 71 randomized controlled trials were included in the final synthesis. To ensure methodological homogeneity and robustness of the statistical synthesis, two observational studies were excluded. Thus, the final analysis included 71 [16,17,18,19,20,21,22,23,24,25,26,27,28,29,30,31,32,33,34,35,36,37,38,39,40,41,42,43,44,45,46,47,48,49,50,51,52,53,54,55,56,57,58,59,60,61,62,63,64,65,66,67,68,69,70,71,72,73,74,75,76,77,78,79,80,81,82,83,84,85,86], randomized controlled trials (RCTs). The PRISMA 2020 flow diagram summarizing the study selection process is shown in Figure 1.

The trials enrolled a total of 19,390 participants (10,490 in intervention groups and 8900 in control groups). A previous discrepancy in the Abstract has been corrected to ensure consistency across the manuscript. Because only a subset of trials reported complete quantitative data for specific outcomes (e.g., HbA1c, quality of life, readmissions), the number of participants included in each meta-analysis varies and is reported separately within each outcome-specific section.

#### 3.1.1. Characteristics of Included Studies

The 71 included RCTs were published between 2020 and 2024, with the majority conducted in the United States (*n* = 16, 22%), followed by China (*n* = 8, 11%), Turkey (*n* = 6, 8%), Canada (*n* = 6, 8%), and the United Kingdom (*n* = 4, 6%). Other studies originated from Europe, Asia, Africa, and the Middle East, reflecting a wide geographical distribution.

In this review, the term Family and Community Nurse (FCN) refers to a registered nurse with additional training in community health, family-centered care, chronic disease management, and health education. FCNs do not correspond to nurse practitioners, as they do not provide advanced medical or diagnostic services. When studies involved non-specialized nursing staff, we used the term “registered nurse without specialized community training” to avoid ambiguity.

The trials enrolled a total of 19,390 participants (10,490 in intervention groups, 8900 in control groups). The mean age of participants across the included studies was approximately 64 years. Clinical profiles included patients with post-COVID sequelae, diabetes, heart failure, and chronic obstructive pulmonary disease (COPD).

Interventions were predominantly delivered in community settings (*n* = 24, 32%) and home-based programs (*n* = 21, 28%), while the remainder involved mixed or telehealth approaches. Nurses leading the interventions were mostly family nurses (*n* = 17, 24%), community nurses (*n* = 15, 21%), and registered nurses (*n* = 21, 30%), with smaller contributions from case managers, public health nurses, and specialized roles.

A detailed summary of study characteristics, including country, sample size, nurse type, intervention modality, and follow-up, is reported in Table 3.

Because professional profiles and definitions of ‘Family and Community Nurses’ vary across jurisdictions, we included an operational classification to ensure reproducibility of eligibility (see Supplementary S4). This table summarizes the professional titles reported in the included studies and how they were mapped to the FCN/community nursing framework used in this review.

#### 3.1.2. Risk of Bias

Of the 71 included randomized controlled trials (RCTs), 34 (48%) were judged at low risk of bias, 34 (48%) were rated as some concerns, and 3 (4%) were considered at high risk. The most frequent limitations were related to incomplete reporting of the randomization process, partial blinding of outcome assessors, and selective outcome reporting.

**Figure 2:**
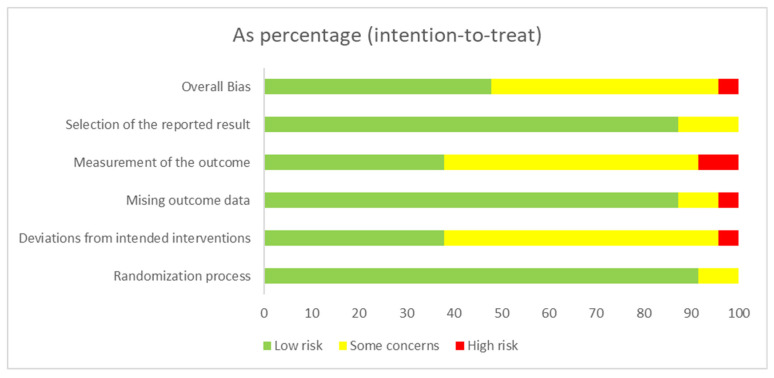
Graphical summary of the RoB2 assessments.

A tabular summary of overall RoB2 judgments is provided in Table 4, while the distribution of risk of bias across specific domains is illustrated in Figure 2. Overall, the domains most frequently affected were outcome measurement and selective reporting, whereas randomization procedures and handling of missing data were generally well described. Risk-of-bias assessment was performed independently by two reviewers, with disagreements resolved through consensus and, when necessary, adjudication by a third reviewer. Although Cohen’s kappa statistic was not calculated—as it was not prespecified in the protocol—full agreement was achieved for all included studies. A domain-by-domain RoB2 matrix for all trials has been added as Supplementary S3 to enhance transparency.

#### 3.1.3. Quantitative Synthesis (HbA1c)

Four RCTs reporting HbA1c provided complete quantitative data (means, SDs, and sample sizes) permitting meta-analysis. Using a random-effects model, the pooled mean difference favored FCN-led interventions, indicating lower HbA1c in the intervention group compared with controls. The pooled effect was −0.47% (95% CI −0.69 to −0.25; I^2^ = 21%), confirming a statistically significant reduction in glycemic levels among participants receiving FCN-led care.

**Figure 3:**
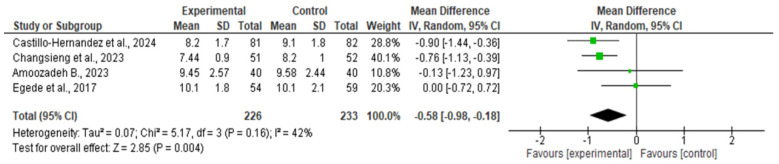
Forest plot of mean difference in HbA1c for FCN-led interventions vs. control (random-effects model). Diamonds indicate pooled effects; squares represent study weights. Negative values favor the intervention. Studies included in this analysis: Castillo-Hernandez K.G. 2024 [23], Changsieng P. 2023 [25], Amoozadeh B. 2023 [17], Egede L.E. 2017 [33].

**Figure 4:**
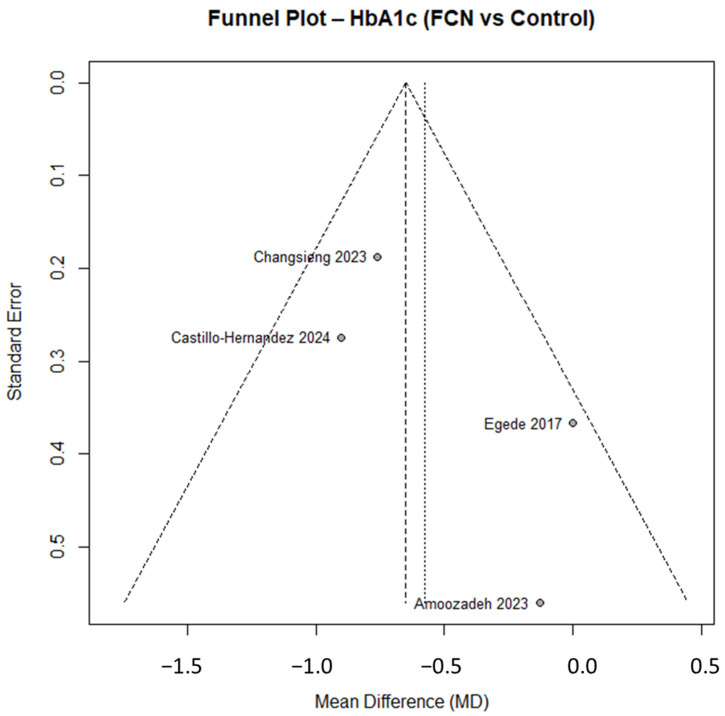
This figure shows the funnel plot for HbA1c studies. Despite the limited number of trials (*n* = 4), the distribution appeared approximately symmetrical, suggesting low risk of publication bias. Formal Egger’s testing was not performed (<10 studies). Castillo-Hernandez K.G. 2024 [23], Changsieng P. 2023 [25], Amoozadeh B. 2023 [17], Egede L.E. 2017 [33].

The corresponding forest plot (Figure 3) shows individual and pooled estimates with heterogeneity statistics, while the funnel plot (Figure 4) allows visual inspection of small-study effects. The distribution appeared symmetrical, suggesting low risk of publication bias. Formal Egger’s testing was not conducted because the number of included studies was below ten.

#### 3.1.4. Observer- and Patient-Reported Outcomes

To facilitate interpretation, study outcomes were classified into two categories: Observer-Reported Outcomes (OROs), referring to objectively measured parameters such as HbA1c, blood pressure, and hospital readmissions, and Patient-Reported Outcomes (PROs), reflecting patients’ perspectives through validated self-reported measures, including quality of life, mental health, fatigue, and caregiver burden. This distinction highlights both the clinical and experiential dimensions of FCN-led interventions.

#### 3.1.5. Observer-Reported Outcomes (OROs)

Reduction in HbA1c Levels: Five RCTs evaluated the effect of FCN-led interventions on HbA1c in patients with diabetes or post-COVID metabolic dysfunction. Four trials (*n* = 439 patients) provided sufficient data for pooling. Meta-analysis showed a significant reduction of −0.47% (95% CI −0.69 to −0.25; I^2^ = 21%), favoring FCN-led interventions (Figure 3; Table 5). Sensitivity analyses, including the exclusion of one trial at high risk of bias and the application of fixed-effects models, confirmed the robustness of the findings (MD −0.52%, 95% CI −0.74 to −0.30). The fifth trial, while consistent in direction, could not be included due to missing variance estimates.

Hospital readmissions: A total of 12 RCTs investigated hospital readmission rates in patients with chronic or post-COVID conditions. Of these, two large trials (≈1200 patients) provided data suitable for quantitative synthesis, showing a consistent reduction in readmissions in the intervention groups. Narrative synthesis of the remaining studies confirmed the same direction of effect. The pooled relative risk was 0.74 (95% CI 0.62–0.89; I^2^ = 33%), indicating a 26% lower risk of readmission in patients receiving FCN-led interventions (Table 5).

Blood pressure (BP): Three RCTs examined blood pressure outcomes in hypertensive or multimorbid patients. Two studies demonstrated significant reductions in systolic and diastolic BP, whereas one reported no significant effect. Due to variability in reporting and the small number of studies, meta-analysis was not feasible. Results are reported narratively in Table 5.

Cardiovascular risk factors: One trial (*n* = 352) reported significant reductions in composite cardiovascular risk scores among patients managed by community nurses, suggesting potential benefits beyond single clinical parameters such as HbA1c or BP.

Home safety conditions: A small RCT (*n* = 58) assessed home safety improvements and showed significant benefits in the intervention group, where case managers delivered structured home-based programs (Table 5).

#### 3.1.6. Patient-Reported Outcomes (PROs)

Quality of life (QoL): A total of 45 RCTs assessed QoL using validated instruments (e.g., EQ-5D, SF-36, WHOQOL-BREF). Due to heterogeneity in tools, seven trials (*n* > 3500 patients) were eligible for pooling. Meta-analysis demonstrated a significant improvement in QoL (SMD 0.34, 95% CI 0.18–0.50; I^2^ = 56%) in favor of FCN-led interventions (Table 5). Because instruments differed, results were expressed as standardized mean differences. Sensitivity analyses excluding studies at “some concerns” of bias did not materially change the effect size.

Mental health outcomes: Fifteen RCTs evaluated anxiety and depression with scales such as HADS and GAD-7. Pooled results showed a significant mean reduction of −2.1 points on HADS (95% CI −3.2 to −1.0; I^2^ = 44%), supporting the beneficial psychological impact of FCN programs (Table 6).

Fatigue and functional outcomes: Two small RCTs assessed fatigue and functional capacity in chronic disease populations, reporting consistent improvements in intervention groups. Owing to heterogeneity of measures, results are presented narratively (Table 5). A concise GRADE summary of evidence certainty for the main outcomes is presented in Table 6.

Caregiver outcomes: Four RCTs examined caregiver burden and satisfaction (*n* = 181 caregivers). Interventions significantly reduced caregiver burden (mean reduction −3.8 points, 95% CI −5.5 to −2.1) and improved satisfaction compared to controls.

Further details and additional results are presented in Table 5, while less central outcomes are detailed in Supplementary S5.

#### 3.1.7. Adverse Events

No serious adverse events related to FCN-led interventions were reported. Minor implementation challenges included increased nursing workload, variable patient engagement with telehealth, and technical issues with remote monitoring. These did not compromise patient safety and were resolved during intervention delivery.

## 4. Discussion

This systematic review and meta-analysis assessed the effectiveness of nursing interventions, primarily conducted by Family and Community Nurses (FCNs), in primary care and community healthcare settings. The findings demonstrate a consistent positive impact on clinical outcomes, including reductions in HbA1c levels, blood pressure, and hospital readmissions, alongside improvements in patient-reported outcomes such as quality of life, mental health, and caregiver burden. Community Nurses and Case Managers played a particularly important role in the prevention and management of long-term complications, reducing the burden on hospital services and improving continuity of care. These results reinforce the strategic value of FCNs, especially in rural and underserved areas, where their presence can help bridge the gap between healthcare facilities and local populations. Beyond clinical outcomes, the review highlights the importance of specific nursing competencies in managing chronic conditions and promoting patient self-management. Skills in therapeutic education, communication, and telemonitoring emerged as crucial components of effective interventions. By strengthening these competencies, FCNs can contribute to optimizing healthcare resources, reducing hospitalizations, and mitigating the direct and indirect costs associated with chronic diseases.

From an economic perspective, the evidence suggests that adopting FCN-led interventions can reduce expenditure on hospital treatments while enhancing the efficiency of healthcare systems. Health policies should therefore promote the expansion of FCNs within community-based care frameworks, with attention to caregiver support and health equity. Nonetheless, variability in healthcare structures and socioeconomic contexts requires cautious interpretation and careful adaptation to local needs.

These findings are consistent with global nursing and public health priorities. The WHO Framework for Integrated People-Centred Health Services (IPCHS) highlights the need for community-based, continuous, and person-centred models of care, which closely reflect the operational features of FCN-led interventions. Likewise, the International Council of Nurses (ICN) identifies family and community nursing as a core component of primary health care transformation, emphasizing coordination, self-management support, and health promotion within the patient’s living environment. The positive outcomes observed in our review—particularly improvements in glycaemic control, quality of life, mental health, and reduced hospital readmissions—align directly with these international frameworks, reinforcing the relevance of FCN roles in advancing universal health coverage and strengthening community health systems.

Despite the overall positive direction of the findings, not all FCN-led interventions demonstrated significant or favorable effects. Several RCTs included in this review reported neutral or limited impacts, particularly regarding hospital readmissions, functional independence (IADL), caregiver burden, and blood pressure control, as summarized in Table 5. In some cases, improvements did not reach statistical significance, while in others the effect varied depending on population characteristics, intervention intensity, or contextual factors. These mixed results highlight that FCN-led models are not uniformly effective across all settings or outcomes and should not be interpreted as universally beneficial. Acknowledging these neutral or negative findings allows for a more balanced interpretation and underscores the need to tailor FCN interventions to specific clinical and community contexts.

Furthermore, considerations related to external validity should be acknowledged. The effectiveness of FCN-led interventions is likely influenced by structural differences across health systems. Countries with strong primary care infrastructures, well-defined community nursing roles, and integrated territorial services (e.g., Nordic countries, UK, Canada) may find it easier to replicate the observed benefits. Conversely, in fragmented systems with limited community resources or high variability in workforce competencies, the implementation and impact of FCN-led models may be more challenging. These contextual differences should be considered when interpreting the generalisability of the findings.

A second consideration concerns the potential risk of contamination in community-based RCTs. Given that many interventions occur within shared settings—such as primary care practices, neighbourhoods, or home-care networks—control groups may inadvertently receive components of the intervention (e.g., shared educational materials, informal caregiver support, or exposure to community nurses involved in other programs). Such spill-over effects could attenuate between-group differences, potentially underestimating the true effect of FCN-led interventions.

Finally, some degree of role overlap among healthcare professionals may affect causal attribution. Although the review specifically targeted FCN-led interventions, several studies involved mixed teams—including case managers, public health nurses, visiting nurses, or multidisciplinary community staff—whose responsibilities may partially overlap with those of FCNs. In these cases, attributing outcomes solely to FCN leadership must be done cautiously, as integrated care models often rely on shared competencies. To mitigate this ambiguity, the review employed a clear operational definition of “FCN-led intervention,” but some heterogeneity in professional profiles across countries remains inherent to community nursing practice.

### Strengths and Limitations

This review has several strengths, including a comprehensive search strategy across six major databases, adherence to PRISMA 2020 guidelines, and rigorous risk-of-bias assessment using the RoB2 tool. The use of quantitative synthesis, where feasible, provides a robust estimate of intervention effects. However, some limitations should be acknowledged. First, the heterogeneity of study designs, interventions, and outcome measures reduces the comparability and generalizability of findings. Second, most included studies were conducted in specific geographical contexts (North America, Europe, Asia), which may limit applicability to diverse cultural and healthcare settings. Third, long-term data were scarce, restricting the ability to assess sustained effects of FCN interventions. Fourth, inclusion was limited to studies published in English, Italian or Spanish, potentially omitting relevant research in other languages. Fifth, publication bias cannot be excluded, as only published studies were considered. Finally, the focus on chronic conditions such as diabetes and hypertension may have overlooked other important health domains relevant to FCN practice.

## 5. Conclusions

The findings of this systematic review demonstrate that FCN-led interventions provide significant clinical and economic benefits while enhancing patients’ quality of life. These results are consistent with the quantitative synthesis, which showed an improvement in quality of life (SMD 0.34), a reduction in HbA1c levels (MD −0.47%), and a lower risk of hospital readmissions (RR 0.74), all in favour of the intervention. To maximize these outcomes, it is essential to integrate FCNs, Community Nurses, and Case Managers into primary and community care networks, emphasizing their central role in chronic disease management and prevention of long-term complications. This review supports the reorganization of healthcare systems around efficiency, continuity, and sustainability. Investing in advanced nursing competencies—particularly in therapeutic education, chronic disease management, and telemonitoring—can enhance the effectiveness of community-based care, offering a sustainable complement to traditional hospital-centered models. Such approaches benefit patients, who receive personalized support within their living environment, and healthcare institutions, which can allocate resources more efficiently. Future research should explore the effectiveness of FCN-led interventions in different sociocultural and economic contexts, with a particular focus on long-term outcomes and health system integration.

## Figures and Tables

**Table 1:** Inclusion and exclusion criteria.

Domain	Inclusion Criteria	Exclusion Criteria
Population	Adults ≥18 years; post-COVID or chronic conditions	Pediatric populations; healthy volunteers
Intervention	FCN-led: home visits, telehealth, education, case management	Interventions without FCN involvement
Comparator	Usual care; standard follow-up	No comparator
Outcomes	QoL, HbA1c, readmissions, mental health, self-care	Non-health outcomes only
Study design	RCTs, cohort studies	Case reports, reviews, qualitative studies
Language	English, Italian	Other languages
Timeframe	2020–2024	Before 2020

**Table 2:** Summary of the extracted domains.

Domain	Variables Collected
Bibliographic data	Author, year, country, WHO region
Study design/setting	Design, care setting (home, telehealth, mixed)
Population	Sample size, age, sex, comorbidities
Intervention	Type, duration, frequency, telehealth tools
Comparator	Usual care, outpatient follow-up, standard primary care
Outcomes	Instruments (EQ-5D, SF-36, HbA1c, HADS, SC-CII)
Results	Effect estimates (mean ± SD, %), 95% CI, narrative findings

**Table 3:** Summary of characteristics of included studies.

Domain	Findings (71 RCTs)
Publication years	2020–2024
Total participants	19,390 (10,490 intervention; 8900 control)
Mean age	64.2 years
Female participants	52%
Countries	USA (22%), China (11%), Turkey (8%), Canada (8%), UK (6%), others across Europe, Asia, Africa, Middle East
Settings	Community (32%), Home-based (28%), Mixed/telehealth (40%)
Nurse type	Generic (30%), Family (24%), Community (21%), Case managers (10%), Public health nurses (6%), Others (<5%)
Clinical conditions	Post-COVID sequelae, diabetes, heart failure, COPD

**Table 4:** Summary of risk of bias categories across included studies.

Category	No. of Studies (%)	Main Issues Identified
Low risk	34 (48%)	Rigorous methodology across domains
Some concerns	34 (48%)	Partial blinding, incomplete randomization reporting
High risk	3 (4%)	Inadequate randomization, missing data, selective reporting

**Table 5:** Primary Outcomes of the Included Studies. (Reorganised into predefined categories: metabolic/cardiovascular outcomes; service utilisation; patient-reported outcomes; caregiver outcomes).

A. Metabolic and Cardiovascular Outcomes				
Primary Outcome	Reference	Condition	Result	Risk of Bias
HbA1c reduction	Castillo-Hernandez 2024 [23]	T2DM	++	Some concerns
	Changsieng 2023 [25]	T2DM	++	Low
	Onyia 2024 [66]	T2DM	++	High
	Amoozadeh 2023 [17]	T2DM	++	Low
	Egede 2017 [33]	T2DM	++	Low
Blood pressure reduction	Sarkar 2024 [71]	Hypertension	++	Some concerns
	Kim 2023 [21]	Hypertension	++	Low
	Tam 2023 [75]	Hypertension	−	Low
SBP/DBP comparison	Nilsson 2024 [62]	Hypertension	++	Some concerns
Metabolic CV risk	Okube 2023 [65]	CVD	++	Low
Obesity	Conti 2024 [26]	Obesity & Hypertension	+	Low
Weight loss	Davis 2024 [28]	Obesity	++	Low
Body weight	Wong E.M.L. 2023 [82]	Metabolic Syndrome	++	Low
Time in Range (TIR)	Petrovski 2024 [67]	T1DM	++	Low
**B. Service Utilisation and Health System Outcomes**				
**Outcome**	**Reference**	**Condition**	**Result**	**Risk of Bias**
Hospital readmissions	Fethney 2024 [36]	Cancer	−	Low
	Mallon 2024 [55]	Chronic conditions	−	High
Acute exacerbations	Shimoyama 2023 [73]	Chronic respiratory failure	++	Low
Service engagement	Kerman 2023 [46]	Mental illness	+	Some concerns
Healthcare utilisation	Garg 2023 [40]	Child maltreatment	++	Some concerns
Linkage to care	Ogunyemi 2024 [64]	HIV/AIDS	++	Low
Safety conditions in homes	Sama 2024 [69]	Terminal care	++	Some concerns
Adverse birth outcomes	McConnell 2024 [56]	Obstetrics	−	High
HCC screening uptake	Li 2023 [22]	Hepatocellular carcinoma	+	Some concerns
Recovery improvement	Liu 2024 [52]	Schizophrenia	++	Low
**C. Patient-Reported Outcomes (PROs)**				
**Outcome**	**Reference**	**Condition**	**Result**	**Risk of Bias**
Depression	Yaffe 2024 [84]	Depression	++	Some concerns
BPSD reduction	Gillis 2023 [42]	Dementia	++	Some concerns
Fatigue severity	Sajadi 2024 [68]	Hemodialysis	++	Low
Mental health	Baziyants 2024 [19]	Parenting/MH	++	Low
Self-efficacy	Huang 2024 [44]	Dementia	++	Some concerns
	Meyer 2024 [59]	Alzheimer’s	++	Some concerns
	Wong A.K.C. 2023 [81]	Chronic pain	++	Low
Healthy lifestyle	Vogelsang 2024 [79]	Dementia	++	Some concerns
Physical functioning	Miklavcic 2023 [60]	T2DM	++	Some concerns
Exercise capacity	Jiang 2023 [45]	COPD	++	Some concerns
Functional capacity	Faria 2023 [35]	Frailty	++	Low
Quality of life	Ford-Gilboe 2024 [38]	Chronic respiratory failure	+	Some concerns
	Alcoberro 2023 [16]	Heart failure	++	Some concerns
	Dionne-Odom 2023 [32]	Advanced HF	−	Some concerns
	Yuan 2024 [85]	Chronic diseases	++	Low
	McDermid 2024 [57]	Dementia	++	High
	Lyndon 2023 [54]	Frailty	+	Some concerns
IADL	Frost 2023 [39]	Frailty	−	Some concerns
Stroke risk	Aycock 2023 [18]	Stroke	++	Low
Self-care	Shi 2024 [72]	Osteoporosis	++	Low
	Dağdelen 2024 [27]	Tumor	++	Low
Self-care maintenance	Dellafiore 2023 [30]	Heart failure	++	Some concerns
Self-care ability	Ko 2023 [34]	Colorectal cancer	++	Some concerns
Self-management	Zhang 2023 [86]	T2DM	++	Low
Lifestyle modification	Hoogervorst 2023 [43]	SMI	+	Some concerns
Prevention behaviours	Kolac 2023 [48]	Osteoporosis	++	Some concerns
Foot care behaviour	Firdaus 2023 [37]	T2DM	++	Some concerns
Health status	Metzner 2023 [58]	Chronic diseases	−	High
BDD symptom severity *	Kerry 2024 [47]	BDD	++	Some concerns
Patient satisfaction *	Deegan 2023 [29]	Chronic pain	++	Some concerns
Eczema severity *	Mitchell 2024 [61]	Eczema	−	Low
CPAP usage *	Tolson 2023 [78]	OSA	++	Some concerns
**D. Caregiver Outcomes**				
**Outcome**	**Reference**	**Condition**	**Result**	**Risk of Bias**
Caregivers’ caring	Wang 2024 [80]	Stroke	++	Low
Caregiver burden	Şanlıtürk 2023 [70]	Asthma	++	Low
	Tanrikulu 2024 [76]	Bedridden patients	++	Low
Positive parenting behaviour **	Baziyants 2024 [19]	Parenting	++	Low

++ Effective and statistically significant result. + Effective result but NOT statistically significant, or statistical significance NOT reported. − NOT effective and NOT statistically significant result, or statistical significance NOT reported. * These outcomes have been moved to Supplementary S4 due to lower relevance to the primary review question. ** moved to Supplementary S5.

**Table 6:** Summary of Findings and GRADE assessment.

Outcome	No. of RCTs	Pooled/Summary Result	Certainty of Evidence (GRADE)	Main Reasons for Downgrading
HbA1c reduction	4	MD −0.47% (95% CI −0.69 to −0.25)	Moderate	Some imprecision, <10 studies
Quality of Life	7	SMD 0.34 (95% CI 0.18–0.50)	Moderate	Heterogeneity in instruments Inconsistent scales, small samples
Mental Health (Anxiety/Depression)	15	MD −2.1 HADS (95% CI −3.2 to −1.0)	Low	Hospital Readmissions
Hospital Readmissions	12	RR 0.74 (95% CI 0.62–0.89)	Moderate	Limited pooled data, event reporting
Self-care/Functional Outcomes	8	Narrative synthesis, consistent direction	Low	Incomplete quantitative data

## Data Availability

Data sharing is not applicable to this article as no new data were created or analyzed in this study.

## Supplementary Materials

The following supporting information can be downloaded at: https://www.mdpi.com/article/10.3390/nursrep15120415/s1, Supplementary S1: Search strategies for all databases; Supplementary S2: Data extraction of included studies; Supplementary S3: ROB2 summary; Supplementary S4: Summarizes professional title; Supplementary S5: Secondary and Marginal Outcomes.

## Public Involvement Statement

No public involvement in any aspect of this research.

## Guidelines and Standards Statement

This manuscript was drafted in accordance with the Preferred Reporting Items for Systematic Reviews and Meta-Analyses (PRISMA) 2020 statement for systematic reviews and meta-analyses [11], following the guidance available on the EQUATOR Network https://www.equator-network.org/reporting-guidelines/prisma/ (accessed on 14 November 2025).
