# Supplementary material for: The Role of the Family and Community Nurse in Improving Quality of Life and Optimizing Home Care Post-COVID: A Systematic Review with Meta-Analysis
Source: Nurs Rep. 2025 Nov 26;15(12):415. doi: 10.3390/nursrep15120415 (PMC12735649; doi:10.3390/nursrep15120415)
Supplement: Supplementary file 1 [file nursrep-15-00415-s001.zip › nursrep-3942326-supplementary_S1.pdf]

Search strategies for all databases

PUBMED:

ARTICLE TYPE

- [ ] Books and Documents
- [x] Clinical Study
- [x] Clinical Trial
- [x] Controlled Clinical Trial
- [ ] Meta-Analysis
- [x] Multicenter Study
- [ ] Articolo di giornale
- [x] Observational Study
- [x] Randomized Controlled Trial
- [ ] Review
- [ ] Systematic Review

PUBLICATION DATE

- (●) 1 year
- ( ) 5 years
- ( ) 10 years
- ( ) Custom Range

SPECIES

- [x] Humans

ARTICLE LANGUAGE

- [x] English
- [x] Italian
- [x] Spanish

Search	Actions	Details	Query	Results	Time
#37	•••	>	Search: ((Nurs*[Title/Abstract]) OR (Family nurse[Title/Abstract])) OR (Community nurse[Title/Abstract])) OR (Public health nurse[Title/Abstract])) OR (Case manager[Title/Abstract])) OR (Advanced practice nurse[Title/Abstract])) OR (District nurse[Title/Abstract])) OR (Home Nurses[Title/Abstract])) AND (Registered nurse[Title/Abstract])) OR (Telemonitoring[Title/Abstract])) OR (Education[Title/Abstract])) OR (Prevention[Title/Abstract])) OR (Primary prevention[Title/Abstract])) OR (Secondary prevention[Title/Abstract])) OR (Tertiary prevention[Title/Abstract])) OR (Direct assistance[Title/Abstract])) OR (Health promotion[Title/Abstract])) OR (Transitional assistance[Title/Abstract])) OR (Discharge planning[Title/Abstract])) OR (Rehabilitation[Title/Abstract])) OR (Intermediate care[Title/Abstract])) OR (Primary healthcare[Title/Abstract])) OR (Basic health care[Title/Abstract])) OR (Community care[Title/Abstract])) OR (Family care[Title/Abstract])) OR (Home care[Title/Abstract])) OR (School*[Title/Abstract])) AND (outcome*[Title/Abstract])) OR (Territory care[Title/Abstract])) OR (Community care[Title/Abstract])) OR (Community[Title/Abstract])) OR (Home based[Title/Abstract])) OR (Home[Title/Abstract])) OR (Territorial assistance[Title/Abstract])) OR (District Care[Title/Abstract])) OR (Care based[Title/Abstract])) OR (Rural assistance[Title/Abstract])) AND (Clinical assistance[Title/Abstract])) OR (Quality of life[Title/Abstract])) OR (Self care[Title/Abstract])) OR (Self management[Title/Abstract])) OR (Self monitoring[Title/Abstract])) OR (Self maintenance[Title/Abstract])) OR (Adherence[Title/Abstract])) OR (Chronicity management[Title/Abstract]) Filters: Clinical Study, Clinical Trial, Controlled Clinical Trial, Multicenter Study, Observational Study, Randomized Controlled Trial, in the last 1 year, Humans, English, Italian, Spanish	5,990	06:06:57

SCOPUS:

Enter query string

(TITLE-ABS-KEY(nurs*) OR TITLE-ABS-KEY(family nurse) OR TITLE-ABS-KEY(community nurse) OR TITLE-ABS-KEY(public health nurse) OR TITLE-ABS-KEY(case manager) OR TITLE-ABS-KEY(advanced practice nurse) OR TITLE-ABS-KEY(district nurse) OR TITLE-ABS-KEY(home nurses) AND TITLE-ABS-KEY(registred nurse) OR TITLE-ABS-KEY(telemonitoring) OR TITLE-ABS-KEY(education) OR TITLE-ABS-KEY(prevention) OR TITLE-ABS-KEY(primary prevention) OR TITLE-ABS-KEY(secondary prevention) OR TITLE-ABS-KEY(tertiary prevention) OR TITLE-ABS-KEY(direct assistance) OR TITLE-ABS-KEY(health promotion) OR TITLE-ABS-KEY(transition assistance) OR TITLE-ABS-KEY(discharge planning) OR TITLE-ABS-KEY(rehabilitation) OR TITLE-ABS-KEY(intermediate care) OR TITLE-ABS-KEY(primary healthcare) OR TITLE-ABS-KEY(basic health care) OR TITLE-ABS-KEY(community care) OR TITLE-ABS-KEY(family care) OR TITLE-ABS-KEY(home care) OR TITLE-ABS-KEY(school*) AND TITLE-ABS-KEY(outcome) OR TITLE-ABS-KEY(territory care) OR TITLE-ABS-KEY(community care) OR TITLE-ABS-KEY(community) OR TITLE-ABS-KEY(home-based) OR TITLE-ABS-KEY(home) OR TITLE-ABS-KEY(territorial assistance) OR TITLE-ABS-KEY(district care) OR TITLE-ABS-KEY(care based) OR TITLE-ABS-KEY(rural assistance) OR TITLE-ABS-KEY(clinical assistance) OR TITLE-ABS-KEY(quality of life) OR TITLE-ABS-KEY(self care) OR TITLE-ABS-KEY(self management) OR TITLE-ABS-KEY(self monitoring) OR TITLE-ABS-KEY(self-maintenance) OR TITLE-ABS-KEY(adherence) OR TITLE-ABS-KEY(chronicity management)) AND PUBYEAR > 2022 AND (LIMIT-TO (LANGUAGE,"English") OR LIMIT-TO (LANGUAGE,"Spanish") OR LIMIT-TO (LANGUAGE,"Italian")) AND (LIMIT-TO (SUBJAREA,"NURS"))

PSYCINFO:

S1	TI (Title: nurs* OR Title: family nurse OR Title: community nurse OR Title: public health nurse OR Title: case manager OR Title: advanced practice nurse OR Title: district nurse OR Title: home nurses AND Title: registred nurse OR Title: telemonitoring OR Title: education OR Title: prevention OR Title: primary prevention OR Title: secondary prevention OR Title: tertiary prevention OR Title: direct assistance OR Title: health promotion OR Title: transition assistance OR Title: discharge planning OR Title: rehabilitation OR Title: intermediate care OR Title: primary healthcare OR Title: basic health care OR Title: community care OR Title: family care OR Title: home care OR Title: shool* AND Title: outcome/* OR Title: territory care OR Title: community care OR Title: community OR Title: home-based OR Title: home OR Title: territorial assistance OR Title: district care OR Title: care based OR Title: rural assistance OR Title: clinical assistance OR Title: quality of life OR Title: self care OR Title: self management OR Title: self monitoring OR Title: self-maintenance OR Title: adherence OR Title: chronicity management) Mostra di meno	**Espansori** - Applica argomenti equivalenti<br>**Modalità ricerca** - Trova tutti i termini della ricerca	Visualizza risultati (120)<br>Modifica
